# Participation in school and physical education in juvenile idiopathic arthritis in a Nordic long-term cohort study

**DOI:** 10.1186/s12969-019-0341-6

**Published:** 2019-07-15

**Authors:** Ellen Nordal, Veronika Rypdal, Ellen Dalen Arnstad, Kristiina Aalto, Lillemor Berntson, Maria Ekelund, Anders Fasth, Mia Glerup, Troels Herlin, Susan Nielsen, Suvi Peltoniemi, Marek Zak, Nils Thomas Songstad, Marite Rygg, for the Nordic Study Group of Pediatric Rheumatology (NoSPeR), Gudmund Marhaug, Freddy Karup Pedersen, Pekka Lahdenne, Boel Anderson-Gäre

**Affiliations:** 1 Department of Pediatrics, University Hospital of North Norway, 9038 Tromsø, Norway; 2 Department of Clinical Medicine, UIT the Arctic University of Norway, Tromsø, Norway; 3 Department of Clinical and Molecular Medicine, NTNU - Norwegian University of Science and Technology, Trondheim, Norway; 4 Department of Pediatrics, Levanger Hospital, Nord-Trøndelag Hospital Trust, Levanger, Norway; 5 Children’s Hospital, University of Helsinki, Helsinki, Finland; 6 Department of Women’s and Children’s Health, Uppsala University, Uppsala, Sweden; 7 Department of Pediatrics, Ryhov County Hospital, Jonkoping, Sweden; 8 Department of Pediatrics, Institute of Clinical Sciences, Sahlgrenska Academy, University of Gothenburg, Gothenburg, Sweden; 9 Department of Pediatrics, Aarhus University Hospital, Aarhus, Denmark; 10 Department of Pediatrics, Rigshospitalet Copenhagen University Hospital, Copenhagen, Denmark; 11 Department of Pediatrics, St. Olavs Hospital, Trondheim, Norway

**Keywords:** Juvenile idiopathic arthritis, Participation, Physical activity, Physical education, School, Epidemiology

## Abstract

**Background:**

The aim of the study was to describe school attendance and participation in physical education in school among children with juvenile idiopathic arthritis (JIA).

**Methods:**

Consecutive cases of JIA from defined geographical areas of Finland, Sweden and Norway with disease onset in 1997 to 2000 were followed for 8 years in a multi-center cohort study, aimed to be as close to population-based as possible. Clinical characteristics and information on school attendance and participation in physical education (PE) were registered.

**Results:**

Participation in school and in PE was lowest initially and increased during the disease course. Eight years after disease onset 228/274 (83.2%) of the children reported no school absence due to JIA, while 16.8% reported absence during the last 2 months due to JIA. Full participation in PE was reported by 194/242 (80.2%), partly by 16.9%, and none by 2.9%. Lowest participation in PE was found among children with ERA and the undifferentiated categories. Absence in school and PE was associated with higher disease activity measures at the 8-year visit. School absence > 1 day at baseline predicted use of disease-modifying anti-rheumatic drugs, including biologics (DMARDs) (OR 1.2 (1.1–1.5)), and non-remission off medication (OR 1.4 (1.1–1.7) 8 years after disease onset.

**Conclusion:**

School absence at baseline predicted adverse long-term outcome. In children and adolescents with JIA participation in school activities is mostly high after 8 years of disease. For the minority with low participation, special attention is warranted to promote their full potential of social interaction and improve long-term outcome.

## Background

Juvenile idiopathic arthritis (JIA) is an umbrella term for chronic childhood arthritis and a significant contributor to chronic disease in children and adolescents. Even in the era of modern treatment with improved outcome on biologic therapies, many children with JIA still experience flares and difficulties to attend daily life activities [1–3].

Children’s right to education is a fundamental human right according to the United Nations [4]. In addition to education, school provides a basic arena for developing a broad range of cognitive and social skills. An increased school absence rate is in general associated with anxiety disorders, increased social isolation, school dropout and lower educational level [5]. Participation is a key issue in the International Classification of Functioning, Disability and Health (ICF), endorsed by the World Health Organization (WHO) [6]. One of the main factors influencing participation is a child’s health condition in addition to personal and environmental determinants, according to the ICF [6]. Physical education in school aims not only to provide physical activity, but also to teach the young person an active life style and increase lifelong physical fitness [7]. All forms of physical activity are now generally recommended and encouraged for children with JIA, replacing the previous traditions of giving advice to avoid certain activities [8]. Limited reports exist on participation in school and in physical education in JIA, and most reports are convenience-sampled, recruited from hospital cohorts, cross-sectional or with short follow-up time, and the studies are often small [3, 9–11].

We have previously performed a longitudinal cohort study of JIA in a population-based setting in the Nordic countries [12]. The data of the Nordic JIA cohort on participation in school and physical education was analyzed in the present study. The main objective was to describe school attendance and participation in physical education during the study period 8 years after disease onset in a population-based setting, and to assess the association with baseline and long-term disease characteristics.

## Methods

### Patients and study design

Children with a follow-up visit 8 years after onset of arthritis were selected to our study on participation in the prospective multicenter Nordic JIA cohort [12, 13]. The original multicenter cohort consisted of children with newly diagnosed JIA according to the International League of Associations for Rheumatism (ILAR) classification criteria, with disease onset in 1997–2000. Pediatric rheumatologists at 12 different centers sent during the period repeated letters to other rheumatologists, orthopedic or pediatric specialists, and the primary health care in their areas, informing about the study and asking for referral of all eligible children. These measures were taken to include all consecutive children diagnosed with JIA from defined geographical area covering large parts of Denmark, Finland, Norway and Sweden, aiming for the cohort to be as close as possible to population-based [12, 13]. Information on school issues were missing for the majority of the Danish participants, and the Finnish patients did not fill in such information until the eight-year visit. Danish participants were therefore excluded from the present study, and Finnish patients were excluded from analyses of the baseline, the one-year and the three-year visits, in order to keep the population-based approach of including all eligible children in the defined geographical areas. Only children in school age in the Nordic countries, defined as 6 years or more of age, with information on school absence and/or physical education were included in analyses at the specific time-points.

### Data acquisition

Clinical information including family history, assessment of disease activity, and self-reported questionnaires were registered per protocol at baseline, at one-year and then at 1–3-year interval and at a pediatric rheumatology study visit collecting more extensive data on remission and damage, approximately 8 years after disease onset [12]. The parent, or parent together with the child if aged ≥9 years, filled in self-reported information on participation in school and physical education. The number of days of school absence due to JIA during the last 2 months before the study visit was reported, including hospital visits and other treatment-related absence. Since the information was collected on a day of a hospital visit, we chose to report the number of children with JIA-related absence of more than 1 day. In the same way we collected self-reported information on full, partly or no participation in physical education class in school. In Scandinavia physical education is an obligatory part of school, usually 1–4 sessions per week, and children with JIA and other chronic diseases may have a “doctor’s excuse” and/or a formally modified alternative physical education program for partial or no participation. The cohort was recently re-examined 18 years after disease onset, but the majority had then finished compulsory school, and at baseline many children were under school-age. The eight-year visit taking place in 2005–2007 was therefore most informative regarding participation in school and PE for the main analyses of this study. We report data at baseline, one-year, three-year and eight-year visits, but because some children had not started school at baseline and some had graduated at the 8-year visit, prediction analyses of baseline school absence on adverse long-term outcome was performed only for the individuals with information available at both time points. The Child Health Assessment Questionnaire (CHAQ) is a validated disease-specific tool to assess functional ability (range 0–3, higher score indicating increasing functional impairment) and was filled in by children aged ≥9 years, otherwise by their parents [14, 15]. The Child Health Questionnaire Parent Form (CHQ-PF50) is a generic tool consisting of 50 items assessing health-related quality of life, yielding a physical summary score (PhS), as well as a psychological summary score (PsS) (range 0–100, lower score indicating impaired functional ability, US healthy reference children have a mean score of 50) [14, 16]. Disease activity was assessed using the Juvenile Arthritis Disease Activity Score (JADAS) [17]. Joint damage was reported using the Juvenile Arthritis Damage Index of articular damage (JADI-A) ranging from 0 to maximum of 72 [18]. Remission was defined according to the preliminary Wallace criteria [19]. Detailed description of the methods of patient accrual and data collection has been previously published [20].

### Statistical analyses

Statistical analyses were performed using the STATA version 15 software (STATA Corp., College Station, TX, USA). Descriptive statistics were used to summarize clinical characteristics of the population and disease activity measures. The chi-square and Fischer’s exact tests were used as appropriate for comparison of dichotomous variables, and the Mann-Whitney U-test for comparison of medians for skewed data. The Student’s *t*-test was used to compare means in continuous outcomes between groups. Univariate logistic regression was performed to assess variables cross-sectionally associated with the outcomes; school absence > 1 day and not participating fully in PE at baseline and at the eight-year visit. Univariate logistic regression was performed to assess school absence > 1 day at baseline as a predictor of adverse outcome in terms of using systemic medication during the disease course, not being in remission and school absence at the eight-year visit only for children that had relevant information available at both timepoints. For variables with *p* < 0.05 in the univariate logistic regression analyses, a multivariable logistic regression was performed adjusting for gender, age and other relevant variables. A *p* value < 0.05 was considered significant.

## Results

Altogether 347 Norwegian, Swedish and Finnish children participated in the Nordic cohort with a long-term follow-up. Among these, 99 Swedish and Norwegian children aged 6 years or more with information on participation in school and/or PE were included in analyses at the baseline visit median 6 (IQR 6–8) months after disease onset, since the Finnish children (*n* = 138) lacked information on participation in the first years of the study, and the Danish children (*n* = 93) lacked all information on participation. The corresponding number of children were 98 at the one-year visit median 13 (IQR 12–15) months, and 125 at the three-year visit median 37 (IQR 36–45) months after disease onset. Among the 347 children with eight-year follow-up, information on participation in school and/or PE was available in 285 (82.1%) children, including Finnish participants at the eight-year visit. Demographic and clinical characteristics of the children with and without information on participation is shown in Table 1. Among the 285 children, 66.0% were female and 50.9% had oligoarticular JIA. The median age was 5.3 (IQR 2.3–9.4) years at onset of disease, and 13.6 (IQR 10.6–17.6) years at the eight-year study visit.

**Table 1:** Clinical characteristics at the eight-year study visit comparing children with juvenile idiopathic arthritis with and without information on physical education (PE) and school attendance

Characteristics at final study visit (*n* = 347)	Participants with information on PE or school attendance (*n* = 285)		Participants without information on PE or school attendance (*n* = 62)	
	*N*	n (%) or median (IQR)	*N*	n (%) or median (IQR)
Female, n (%)	285	188 (66.0)	62	39 (62.9)
Age at disease onset, years	285	5.3 (2.3–9.4)	62	8.3 (3.8–11.2)
Cumulative joint count	285	6 (2–12)	62	4 (2–10)
Active joints	285	0.0 (0.0–0.0)	62	0.0 (0.0–0.0)
Physician GA, VAS	192	0.0 (0.0–0.9)	4	0.3 (0.1–1.8)
Patient/parent GA, VAS	216	0.0 (0.0–1.2)	3	0.3 (0.0–0.5)
Pain VAS	220	0.0 (0.0–1.3)	3	0.0 (0.0–0.5)
CHAQ score	285	0.0 (0.0–0.1)	8	0.0 (0.0–0.1)
CHQ-PF50, PhS score	160	52.5 (45.5–56.9)	4	46.8 (36.2–53.9)
CHQ-PF50, PsS score	160	54.8 (48.0–59.6)	4	40.9 (32.4–46.8)
DMARDs, n (%)	285	102 (35.8)	62	8 (12.9)
Oligoarticular persistent, n (%)	285	84 (29.5)	62	25 (40.3)
Oligoarticular extended, n (%)	285	49 (17.2)	62	8 (12.9)
Polyarticular RF negative, n (%)	285	58 (20.3)	62	7 (11.3)
Polyarticular RF positive, n (%)	285	2 (0.7)	62	1 (1.6)
Systemic arthritis, n (%)	285	8 (2.8)	62	1 (1.6)
Psoriatic arthritis, n (%)	285	13 (4.6)	62	0 (0)
Enthesitis-related arthritis, n (%)	285	29 (10.2)	62	12 (19.3)
Undifferentiated arthritis, n (%)	285	42 (14.7)	62	8 (12.9)
ESR mm/hour	193	7.0 (4.0–11.0)	14	8.5 (6.0–14.0)
CRP mg/liter	201	0.0 (0.0–5.0)	12	2.5 (0.0–7.5)
RF positive, n (%)	150	5 (3.3)	29	2 (6.9)
HLA-B27 positive, n (%)	274	62 (22.6)	52	11 (21.2)
ANA positive, n (%)	284	66 (23.2)	60	13 (21.7)

Values are the median (interquartile range, IQR), unless indicated otherwise. *PE* Physical education and school attendance assessed at the eight-year study visit, *GA* Global assessment, *VAS* Visual analogue scale, *CHAQ* Childhood health assessment questionnaire, *CHQ-PF50* Child health questionnaire parent form, *PhS* Physical summary score, *PsS* Psychological summary score, *DMARDs* Disease-modifying antirheumatic drugs including biologics used at the final study visit, *ESR* Erythrocyte sedimentation rate, *CRP* C-reactive protein, *ANA* Antinuclear antibody, assessed twice with at least 3 months interval, *RF* Rheumatoid factor, assessed twice with at least 3 months interval, *HLA-B27* Human leucocyte antigen

### School attendance

**Fig. 1:**
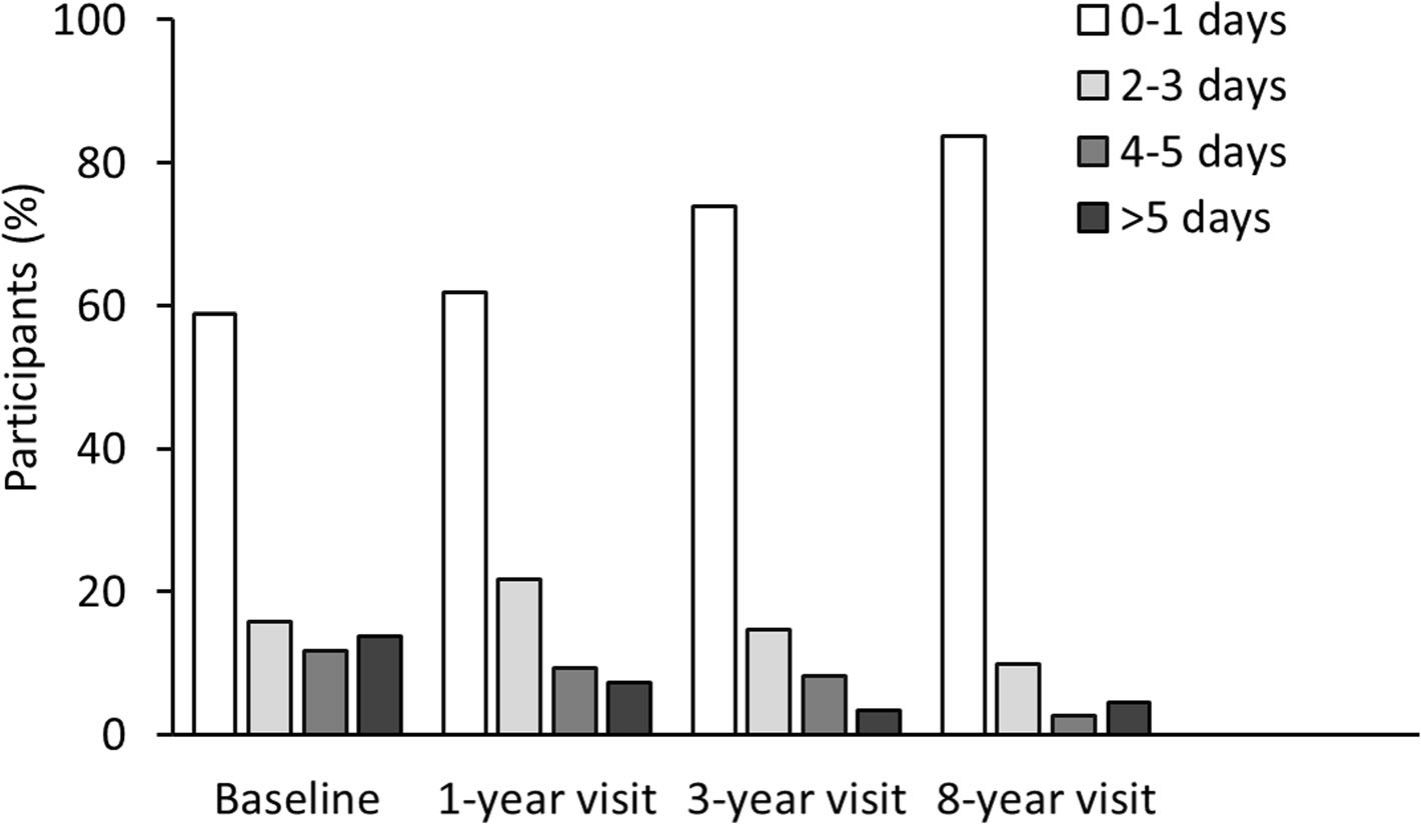
Number of days of school absence because of JIA during the last 2 months among participants in the Nordic JIA study. **a** assessed at the baseline study visit 7 months after disease onset (*n* = 95). **b** assessed at the eight-year study visit (*n* = 274)

Mean school absence during the last 2 months because of JIA was 2.7 days at baseline, 1.7 days at the one-year visit, and 1.5 days at the three-year visit. At baseline 56/95 (58.9%) children reported no school absence because of JIA within the last 2 months before the study visit, 39 (41.1%) reported more than 1 day, 24 (25.3%) reported more than 3 days, and 13 (13.7%) reported more than 5 days, as shown in Fig. 1. No school absence because of JIA was reported by 60 (61.9%) children at the one-year visit, and by 90 (73.8%) at the three-year visit (Fig. 1). Eight years after disease onset, 228/274 (83.2%) reported no school absence because of JIA, while 46 (16.8%) reported more than 1 day of absence, 18 (6.9%) reported more than 3 days and 12 (4.4%) reported more than 5 days (Fig. 1). School absence because of JIA within the last 2 months before the eight-year study visit was mean 1.1 days. In Table 2 the characteristics of the cohort are shown according to school absence 8 years after disease onset. There was no impact of gender, age at visit, nor BMI on school attendance. There were no significant differences in school attendance between JIA categories (*p* = 0.078, Fischer’s exact test, results not shown). Children with more than 1 day absence had significantly lower CHQ physical summary score (41.8 (IQR 32.1–50.4)) than children with full school attendance (53.4 (IQR 47.9–57.3)), while there were no differences in CHQ psychosocial summary score (Table 2).

**Table 2:** Characteristics of 274 children in the Nordic JIA cohort according to school absence at the eight-year study visit^a^

Characteristics at the eight-year visit	n	Absence ≤1 day (*n* = 228)	Absence > 1 day (*n* = 46)	OR (95% CI)	*P*-value
Female, n (%)	274	148 (64.9)	32 (69.6)	1.2 (0.6–2.4)	0.545
Age disease onset, years	274	5.2 (2.3–9.2)	4.6 (2.3–8.7)	1.0 (0.9–1.1)	0.440
BMI	234	19.5 (17.3–21.6)	19.8 (17.2–24.8)	1.1 (1.0–1.2)	0.117
Cumulative joint count	274	5 (2–10)	11 (5–14)	1.0 (1.0–1.0)	0.007
Active joints	274	0 (0–0)	0 (0–1)	1.5 (1.2–1.9)	0.002
Physician GA	190	0.0 (0.0–0.5)	1.0 (0.2–2.1)	7.1 (2.7–18.5)	< 0.001
Patient/parent GA	213	0.0 (0.0–0.6)	1.2 (0.0–2.5)	3.5 (1.8–6.8)	< 0.001
Pain VAS	217	0.0 (0.0–0.8)	1.4 (1.0–3.5)	3.5 (1.9–6.5)	< 0.001
CHAQ	274	0.0 (0.0–0.0)	0.3 (0.0–0.6)	10.1 (4.4–23.1)	< 0.001
CHQ-PF50, PhS	159	53.4 (47.9–57.3)	41.8 (32.1–50.4)	0.9 (0.8–0.9)	< 0.001
CHQ-PF50, PsS	159	54.9 (47.5–60.1)	54.3 (49.7–58.4)	1.0 (0.9–1.1)	0.792
DMARDs, n (%)	274	72 (31.6)	27 (58.7)	1.8 (1.3–2.7)	0.002
JADAS10	174	0.4 (0.0–2.2)	3.5 (0.6–6.1)	1.3 (1.2–1.5)	< 0.001
Not in remission, n (%)	267	126 (56.8)	42 (93.3)	10.7 (3.2–35.5)	< 0.001
ESR mm/hour	193	7.0 (4.0–11.0)	8.0 (4.0–13.0)	1.0 (1.0–1.1)	0.110
CRP mg/liter	201	0.0 (0.0–5.0)	0.0 (0.0–5.0)	1.0 (1.0–1.1)	0.168
RF positive	147	3 (2.6)	2 (6.3)	2.5 (0.4–15.6)	0.330
HLA-B27 positive	264	45 (20.6)	15 (32.6)	1.9 (0.9–3.7)	0.081
ANA positive	273	57 (25.1)	7 (15.2)	0.5 (0.2–1.3)	0.154

^a^Patient-/ proxy-reported school absence because of JIA during the preceding 2 months of the eight-year study visit with univariate logistic regression. Values are the median (interquartile range, IQR), unless indicated otherwise, *OR* Odds ratio, *CI* Confidence interval, *GA* Global assessment, *VAS* Visual analogue scale, *CHAQ* Childhood health assessment questionnaire, *CHQ-PF50* Child health questionnaire parent form, *PhS* Physical summary score, *PsS* Psychological summary score, *DMARDs* Disease-modifying antirheumatic drugs including biologics used at the final study visit, *Not in remission* Not in remission off medication according to the Wallace preliminary criteria [21], *ESR* Erythrocyte sedimentation rate, *CRP* C-reactive protein, *RF* Rheumatoid factor, assessed twice with at least 3 months interval, *HLA-B27* Human leucocyte antigen, *ANA* Antinuclear antibody, assessed twice with at least 3 months interval

### Participation in physical education in school

**Fig. 2:**
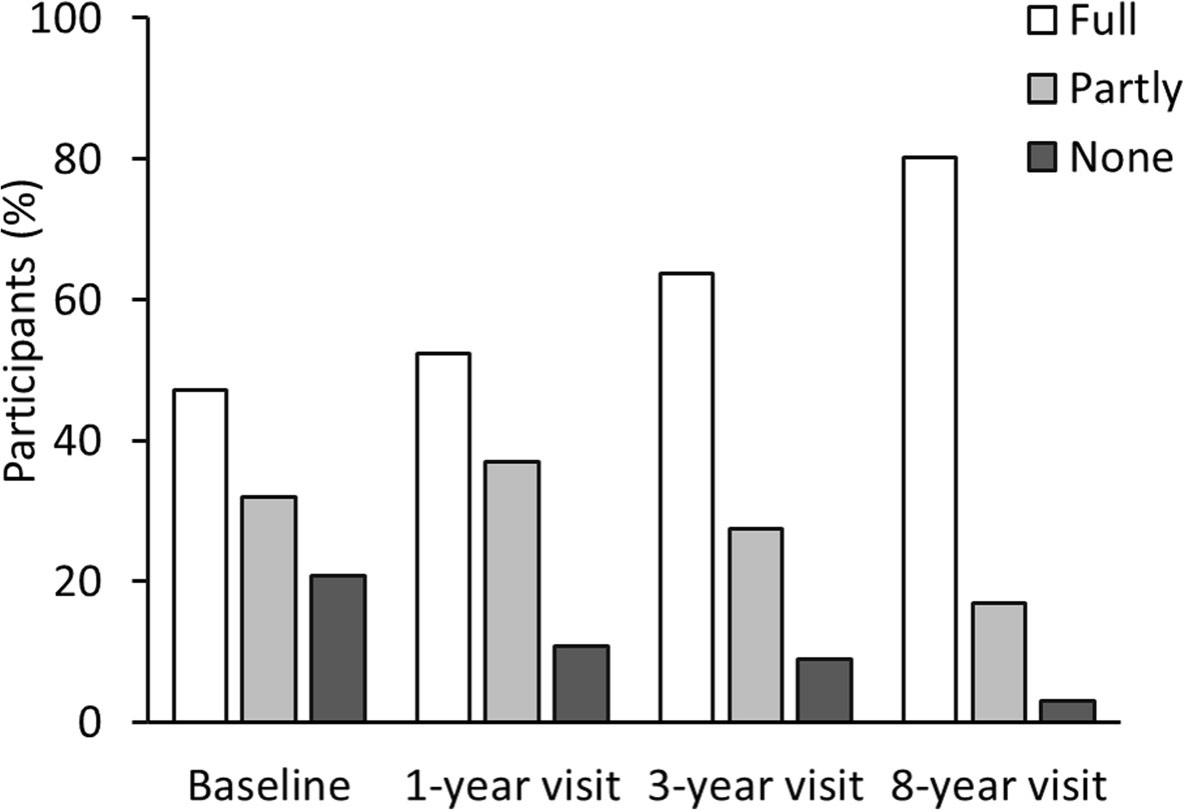
Participation in physical education in school within the last 2 months before the baseline study visit reported 7 months after disease onset (*n*=89), at the 1-year visit (*n*=84), the 3-year visit (*n*=113), and the 8-year visit (*n*=242)

At baseline 6 months after disease onset 44/89 (49.4%) reported full participation in PE, partly participation was reported by 33 (37.1%), and 12 (13.5%) did not participate, as shown in Fig. 2. Full participation increased over time to 52.4% of the children at the one-year visit and 63.7% at the three-year visit. Eight years after disease onset full participation in PE was reported by 194/242 (80.2%), partly participation by 41 (16.9%), and 7 (2.9%) reported no participation (Fig. 2). Table 3 shows the characteristics of PE participation at the eight-year visit. Significantly fewer girls than boys participated fully in PE (*p* = 0.01). Higher BMI (*p* = 0.01) were associated with less participation in PE. Children that did not participate fully in physical education had significantly lower CHQ physical and also psychosocial summary scores, as shown in Table 3. Fewer children participated fully in PE in the enthesitis-related arthritis (ERA) (65.0%) and the undifferentiated (61.8%) categories, while participation was highest in the oligoarticular persistent category (93.6%) (Table 4). Among the 46 children with school absence more than 1 day at the eight-year visit, 22/41 (53.7%) did not participate or participated only partly in physical education. Information on participation in PE was missing in five of these children.

**Table 3:** Characteristics of 242 children in the Nordic JIA cohort according to PE participation at the 8 year visit^a^

Characteristics at the eight-year visit	Total n	Full PE (*n* = 194)	Partly or no PE (*n* = 48)	OR (95% CI)	*P*-value
Females, n (%)	242	119 (61.3)	39 (81.3)	2.7 (1.3–6.0)	0.012
Age at disease onset, years	242	4.4 (2.2–7.3)	4.6 (2.3–8.7)	1.0 (0.9–1.1)	0.398
BMI	205	18.9 (16.8–21.0)	21.2 (17.8–23.5)	1.1 (1.0–1.2)	0.013
Cumulative joint count	242	5 (2–9)	12 (4–16)	1.1 (1.0–1.1)	< 0.001
Active joints	242	0 (0–0)	0 (0–2)	2.0 (1.4–2.8)	< 0.001
Physician GA, VAS	188	0.0 (0.0–0.5)	0.9 (0.2–2.0)	7.1 (2.7–18.8)	< 0.001
Patient/parent GA, VAS	212	0.0 (0.0–0.4)	1.6 (0.3–3.4)	6.1 (2.9–12.6)	< 0.001
Pain VAS	216	0.0 (0.0–0.5)	2.0 (0.8–5.6)	7.8 (3.8–15.9)	< 0.001
CHAQ score	242	0.0 (0.0–0.0)	0.3 (0.0–1.0)	15.0 (5.9–38.6)	< 0.001
CHQ-PF50, PhS score	156	53.9 (48.5–57.3)	41.5 (31.1–48.2)	0.9 (0.8–0.9)	< 0.001
CHQ-PF50, PsS score	156	56.0 (49.9–60.1)	50.3 (44.6–55.8)	0.9 (0.9–1.0)	0.010
DMARDs, n (%)	242	64 (33.0)	24 (50.0)	1.5 (1.1–2.2)	0.025
JADAS10	147	0.1 (0.0–1.9)	4.7 (1.9–8.0)	1.5 (1.3–1.7)	< 0.001
Not in remission, n (%)	236	106 (55.8)	42 (91.3)	6.5 (2.5–17.2)	< 0.001
ESR mm/hour	164	7.0 (4.0–10.0)	12.0 (5.0–16.0)	1.1 (1.0–1.1)	0.029
CRP mg/liter	169	0.0 (0.0–5.0)	0.0 (0.0–5.0)	1.0 (0.9–1.0)	0.851
RF positive, n (%)	123	1.0 (1.1)	0	n.a	n.a
HLA-B27 positive, n (%)	233	42 (22.6)	10 (21.3)	0.9 (0.4–2.0)	0.848
ANA positive, n (%)	241	51 (26.4)	8 (16.7)	0.6 (0.2–1.3)	0.164

^a^Patient-/ proxy-reported participation in physical education due to JIA during the preceding 2 months of the eight-year study visit, univariate logistic regression. Values are the median (interquartile range, IQR), unless indicated otherwise, *OR* Odds ratio, *CI* Confidence interval, *GA* Global assessment, *VAS* Visual analogue scale, *CHAQ* Childhood health assessment questionnaire, *CHQ-PF50* Child health questionnaire parent form, *PhS* Physical summary score, *PsS* Psychological summary score, *DMARDs* Disease-modifying antirheumatic drugs including biologics used at the final study visit, *Not in remission* Not in remission off medication according to the Wallace preliminary criteria [21], *ESR* Erythrocyte sedimentation rate, *CRP* C-reactive protein, *RF* Rheumatoid factor, assessed twice with at least 3 months interval, *n.a.* Not applicable, *HLA-B27* Human leucocyte antigen, *ANA* Antinuclear antibody, assessed twice with at least 3 months interval

**Table 4:** Participation in physical education (PE) in 242 children at the eight-year study visit in the Nordic juvenile idiopathic arthritis (JIA) cohort according to different JIA categories

JIA categories	Full PE (*n* = 194)	No or partly PE (*n* = 48)	OR (95% CI)^a^	*P*-value
	n (%)	n (%)		
Oligoarticular persistent	73 (93.6)	5 (6.4)	n.a	n.a
Oligoarticular extended	33 (78.6)	9 (21.4)	4.0 (1.2–12.8)	0.020
Polyarticular RF negative	39 (78.0)	11 (22.0)	4.1 (1.3–12.7)	0.014
Polyarticular RF positive	0	0	n.a	n.a
Systemic arthritis	5 (83.3)	1 (16.7)	2.9 (0.3–30.0)	0.367
Psoriatic arthritis	10 (83.3)	2 (16.7)	2.9 (0.5–17.1)	0.235
Enthesitis-related arthritis	13 (65.0)	7 (35.0)	7.9 (2.2–28.6)	0.002
Undifferentiated arthritis	21 (61.8)	13 (38.2)	9.0 (2.9–28.3)	< 0.001

^a^Univariate regression analysis with oligoarticular persistent arthritis as a reference category. *OR* Odds ratio, *CI* Confidence interval, *RF* Rheumatoid factor, *n.a* Not applicable

### Associations between participation and disease characteristics

In univariate analyses, lower school attendance at the eight-year visit was significantly associated with higher number of active and cumulative joints, higher level of pain and global disease impact on overall well-being assessed by the child/parent, higher global disease severity assessed by the physician, higher CHAQ score, lower physical summary score of the CHQ, higher JADAS10 score, ongoing DMARDs, and not being in remission off medication (Table 2). These associations remained significant after adjusting for gender and age at the 8-year visit in a multivariable logistic regression model (results not shown).

All characteristics associated with school absence mentioned above, were also associated with less participation in PE (Table 3), and these associations remained significant when adjusting for gender and age at the 8-year visit in a multivariable logistic regression model (results not shown).

School absence more than 1 day the preceding 2 months before the baseline visit predicted higher school absence at the eight-year visit (OR 1.2 (1.0–1.4), *p* = 0.044) in the 92 children with both baseline and eight-year information available. School absence at baseline did also predict use of disease-modifying anti-rheumatic drugs, including biologics (DMARDs) (OR 1.2 (1.1–1.5), *p* = 0.011, *n* = 97), during the eight-year follow-up period. School absence more than 1 day at baseline was also a significant predictor of not being in remission off medication (OR 1.4 (1.1–1.7), *p* = 0.007, *n* = 94) in a univariate model. School absence at baseline remained significant for predicting the use of DMARDs (OR 1.2 (1.0–1.5)) and for not achieving remission (OR 1.3 (1.0–1.6)) when adjusting for gender, age, and the number of active joints during the first 6 months in a multivariable regression model (results not shown).

## Discussion

We assessed school attendance and participation in physical education in a prospective multi-center Nordic JIA cohort. School attendance increased during the disease course, and was generally high in children 8 years after disease onset in the Nordic JIA cohort. Participation in PE in school was also high, but we found less participation in PE in the ERA and undifferentiated categories. Higher pain scores and disease activity measures were significantly associated with higher school absence and less participation in PE at the eight-year visit. Moreover, school absence at baseline predicted use of DMARDs during the disease course, and not being in remission 8 years after disease onset.

We did not find any other reports showing that baseline school absence predicts adverse outcomes, such as the need for DMARDs including biologic agents during the disease course and not being in long-term remission off medication. This finding was robust for adjustments for gender, age, and number of active joints in early disease, pointing to early school absence as a warning sign of a more severe disease over time.

Our results indicate increased school absence in children with JIA compared to healthy school children, since the participants were asked to report JIA-related school absence. We did not ask for school absence not related to JIA. In Denmark, national statistics show a mean of 5.0% school absence in all school children [22]. Sixteen-year old Norwegian students have a mean absence of 3 days and 9 h per school year [23]. This corresponds to 2 days (Denmark) or half a day (Norway) per two-month period, in comparison to the reported additional 2.7 days at baseline and 1.1 days at the eight-year visit of absence related to JIA in our study.

A lower attendance rate was found in children with JIA in a British cross-sectional study. Sturge et al. reported a mean school attendance rate for children with JIA of 92%, equivalent to 15 yearly absent days [24]. Attendance was lowest in the more severely affected polyarticular JIA category in this study published in 1997 before the era of biologic treatment [24]. In a Moroccan study Bouaddi et al. reported that as many as 33% of children with JIA were unable to attend school due to their disease. In line with our results the risk of increasing school absence was clearly associated to higher disease activity [25], which is not uniformly reported in all recent studies [26]. JIA may have a higher impact on participation in less privileged countries, due to limited access to expensive medical treatment [27]. Cultural and social traditions in coping with chronic childhood diseases may also contribute to differences in different regions of the world. Few data are available to compare school participation in JIA to other chronic childhood diseases. In the Swedish diabetes registry Persson et al. report that type 1 diabetes had a small, but significant, negative effect on schooling in terms of school grades, and opportunities for further education and careers [28]. Nilsson et al. found indications that asthma may impair school performance in adolescence in a population-based birth cohort [29]. In comparison, Mease et al. reported that adults with ankylosing spondylitis missed an average 6.3% of work time due to a disease-related problems [30]. School absence is associated with increased school dropout, and the critical years for decisions on education may coincide with the period of transfer between pediatric and adult rheumatology clinics with increased risk of disease flares and lower health-related quality of life [31, 32]. In a systematic literature review young adults with arthritis had a higher rate of unemployment compared to healthy peers [21], underlining that school absence, social participation, educational and vocational needs are central issues to address in transitional care [32, 33]. Further follow-up of our cohort regarding transition, educational level and employment rates are planned, but not yet available.

A majority of the children in our cohort participated fully in physical education, and only 2.9% reported no participation. In two recent reports from the Nordic countries both Risum et al. and Nørgaard et al. found similarly high participation rates in physical education for children and adolescents with JIA in, respectively, a Norwegian and a Danish controlled cross-sectional study [10, 11]. In a German registry study Milatz et al. found that 66% of 5879 school-aged children with JIA participated fully in school sports, while 16% did not participate [34]. We found less participation in PE in ERA and the undifferentiated JIA category, in the latter many had ERA-associated characteristics [10, 25]. An explanation can be that ERA and undifferentiated arthritis are shown to have higher pain scores, and higher disability than other JIA categories. Also, adolescents with ERA had a lower rate of remission, both in our cohort and in other outcome studies [12, 35–37]. Physical activity is especially recommended for adult spondyloarthropathies [38, 39]. Regular physical activities have an impact on muscular strength, physical and cardiovascular capacity, as well as psychosocial health [40, 41]. There is evidence that structured aerobic and neuromuscular training may improve exercise capacity, functional performance, and quality of life in children and adolescents with JIA [42, 43], and in adults with rheumatic inflammatory diseases [44]. A positive association between increased bone mass and physical activity during the life course, and particularly in the growth period, is found in a literature review [45]. Recent research also points to an anti-inflammatory effect of exercise that may be mediated by muscle-derived cytokines [9, 46]. Armbrust el al examined effect of an internet program to promote physical activity in children with JIA, and report that full participation in physical education increased from 57 to 71% in their intervention group of 28 children [42]. This knowledge underlines the need to provide satisfactory tailored physical education programs to children with JIA that do not take part in PE.

The prospective, observational cohort design and population-based context are the strengths of this multicenter cohort study. The proportion of children lost to follow-up is small compared to other longitudinal studies [47–49]. A novelty is that our study can show school attendance and participation development longitudinally during the disease course, assess associations of participation to patient and disease characteristics, and also assess baseline school absence as a predictor of adverse outcome. Since this information was collected at a hospital visit, we report “more than one of day absence” as absence because of JIA. Very few patients will have more than this 1 day spent for hospital visits during the last 2 months, which is the period we asked for. A weakness is that there were no control group, and not all of the children in our cohort had information on school and physical education at all four time-points during the follow-up period. Recall bias may have influenced the data, since participation during the previous 2 months was registered by the child or the parents at a study visit. Asking for school absence related to JIA also leaves the judgment to the individual participants as to whether intercurrent infectious diseases or psychosocial problems were considered related to JIA or not.

## Conclusion

Participation in school and PE is high in this eight-year follow-up of the Nordic JIA cohort, but lower participation in PE was found among children with ERA and the undifferentiated categories. Increased school absence at baseline predicted long-term adverse outcome in terms of use of DMARDs including biologic agents and not achieving remission off medications.

Increased school absence and less participation in physical education 8 years after disease onset was also significantly associated with higher disease activity, functional disability and lower quality of life-measures. Attention is warranted to ongoing promotion of physical activity in children with JIA. School attendance needs continuous focus in JIA follow-up, especially in the vulnerable transition period for adolescents.

## Data Availability

The datasets generated and/or analyzed during the current study are not publicly available for ethical reasons, as well as privacy reasons, but are available from the Nordic Study group of Pediatric Rheumatology (NoSPeR) on reasonable request.

## Abbreviations

ANA: Antinuclear antibodies
BMI: Body mass index
CHAQ: Childhood Health Assessment Questionnaire
CHQ PhS: Child Health Questionnaire Physical summary score
CHQ PsS: Child Health Questionnaire Phychosocial summary score
CHQ-PF56: Child Health Questionnaire-Parent Form 56
CI: Confidence interval
CRP: C-reactive protein
DMARD: Disease-modifying antirheumatic drugs
ERA: Enthesitis-related arthritis
ESR: Erythrocyte sedimentation rate
GA: Global assessment
HLAB27: Human leucocyte antigen B27
ICF: International Classification of Functioning, Disability and Health
ILAR: International League of Associations for Rheumatology
IQR: Interquartile range
JADAS: Juvenile Arthritis Disease Activity Score
JADI-A: Juvenile Arthritis Damage Index, Articular Score
OR: Odds ratio
PE: Physical education
RF: Rheumatoid factor
VAS: Visual analogue scale
WHO: World Health Organization
